# The Metabolomic Effects of Tripeptide Gut Hormone Infusion Compared to Roux-en-Y Gastric Bypass and Caloric Restriction

**DOI:** 10.1210/clinem/dgab608

**Published:** 2021-08-30

**Authors:** Ben Jones, Caroline Sands, Kleopatra Alexiadou, James Minnion, George Tharakan, Preeshila Behary, Ahmed R Ahmed, Sanjay Purkayastha, Matthew R Lewis, Stephen Bloom, Jia V Li, Tricia M Tan

**Affiliations:** 1 Section of Endocrinology and Investigative Medicine, Department of Metabolism, Digestion and Reproduction, Faculty of Medicine, Imperial College London, London, UK; 2 National Phenome Centre, Department of Metabolism, Digestion and Reproduction, Faculty of Medicine, Imperial College London, London, UK; 3 Department of Surgery and Cancer, Imperial College Healthcare NHS Trust, London, UK; 4 Division of Digestive Diseases, Department of Metabolism, Digestion and Reproduction, Faculty of Medicine, Imperial College London, London, UK

**Keywords:** GLP-1, oxyntomodulin, PYY, bariatric surgery, caloric restriction, metabolomics

## Abstract

**Context:**

The gut-derived peptide hormones glucagon-like peptide-1 (GLP-1), oxyntomodulin (OXM), and peptide YY (PYY) are regulators of energy intake and glucose homeostasis and are thought to contribute to the glucose-lowering effects of bariatric surgery.

**Objective:**

To establish the metabolomic effects of a combined infusion of GLP-1, OXM, and PYY (tripeptide GOP) in comparison to a placebo infusion, Roux-en-Y gastric bypass (RYGB) surgery, and a very low-calorie diet (VLCD).

**Design and Setting:**

Subanalysis of a single-blind, randomized, placebo-controlled study of GOP infusion (ClinicalTrials.gov NCT01945840), including VLCD and RYGB comparator groups.

**Patients and Interventions:**

Twenty-five obese patients with type 2 diabetes or prediabetes were randomly allocated to receive a 4-week subcutaneous infusion of GOP (n = 14) or 0.9% saline control (n = 11). An additional 22 patients followed a VLCD, and 21 underwent RYGB surgery.

**Main Outcome Measures:**

Plasma and urine samples collected at baseline and 4 weeks into each intervention were subjected to cross-platform metabolomic analysis, followed by unsupervised and supervised modeling approaches to identify similarities and differences between the effects of each intervention.

**Results:**

Aside from glucose, very few metabolites were affected by GOP, contrasting with major metabolomic changes seen with VLCD and RYGB.

**Conclusions:**

Treatment with GOP provides a powerful glucose-lowering effect but does not replicate the broader metabolomic changes seen with VLCD and RYGB. The contribution of these metabolomic changes to the clinical benefits of RYGB remains to be elucidated.

Type 2 diabetes (T2D) affects 1 in 10 people and is responsible for 4.2 million deaths every year (1), as well as severe complications including cardiovascular disease, retinopathy, nephropathy, and amputations (2). The increasing prevalence of obesity drives a parallel increase in T2D due to the shared pathophysiological processes of these 2 metabolic diseases (3). Bariatric surgery is the most effective treatment for both morbid obesity and T2D, significantly outperforming lifestyle interventions and pharmacotherapy (4). One proposed mechanism for the beneficial metabolic effects of bariatric surgery is the observed increase in postprandial release of glucoregulatory and satiety gut hormones including glucagon-like peptide-1 (GLP-1), oxyntomodulin (OXM), and peptide YY (PYY) (5). To probe this, we have previously administered these 3 gut hormones in combination (tripeptide GOP) as a daily subcutaneous infusion for 4 weeks in overweight patients with T2D (6) to achieve circulating concentrations comparable to peak levels seen after Roux-en-Y gastric bypass (RYGB) surgery. In fact, tripeptide GOP infusion led to better improvements in glycemia compared to parallel groups undergoing RYGB or caloric restriction [very low-calorie diet (VLCD)], despite less weight loss. This implies that the tripeptide infusion exerts a potent effect on glucose homeostasis that is at least partly independent of weight loss (6) and also provides further evidence that other mechanisms beyond elevations in gut hormones may be operative after RYGB.

Metabolic profiling analyses based on proton nuclear magnetic resonance (^1^H NMR) spectroscopy and mass spectrometry have made investigation of the biochemically diverse human metabolome and lipidome accessible, helping further the understanding of normal physiology and numerous disease states (7). A number of previous studies have demonstrated that both RYGB (8-16) and VLCD (17-21) result in profound changes to the plasma and urinary metabolome. Certain features suggest common underlying metabolic processes, such as increases in plasma ketone derivatives, fatty acids, and acylcarnitine species as responses to caloric restriction (22). RYGB appears to exert additional effects such as an early reduction (within weeks) in circulating branched-chain amino acids (BCAAs), a change that is suggested by some to exert beneficial effects on glucose metabolism (23-25). However, a recent study showed that the glucose-lowering effects of bariatric surgery are not dependent on a reduction in BCAAs (26). Moreover, some metabolomic changes apparently specific to RYGB may in fact be due to a preoperative period of caloric restriction, which is commonly recommended prior to the procedure (27). The nature and importance of bariatric surgery-associated metabolomic changes are therefore unresolved. Furthermore, the effect of combined gut hormone infusion on the metabolome is not known, although effects of GLP-1 receptor agonist treatment on lipid and lipoprotein parameters have been reported in the literature (28,29).

With the aim of investigating potential shared or distinct mechanisms through which GOP, RYGB, and VLCD achieve their glucoregulatory and weight lowering benefits, we provide in this study comprehensive analyses of the effects of a 4-week infusion of GOP or vehicle control [saline (SAL)], RYGB, and VLCD on the circulating and urinary metabolome. In our study patients that underwent RYGB did not follow a VLCD prior to the procedure, allowing us to discount this potential confounder. Overall, we observed a limited impact on the metabolome of tripeptide GOP infusion, contrasting with the wide-ranging and generally well-correlated effects of both VLCD and RYGB.

## Materials and Methods

### Study Design and Participants

**Figure 1.**
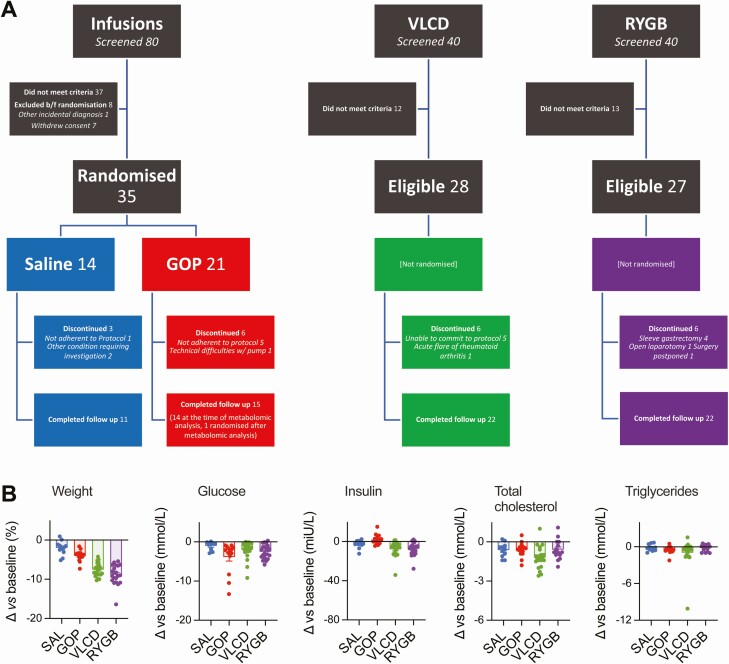
CONSORT diagram and summary of effects of each intervention on clinical parameters. (A) CONSORT flow chart. Note that 1 patient in the original GOP group was randomized after the metabolomic analysis for other participants was completed and is not included in the current manuscript. (B) Changes in clinical parameters from baseline for each individual.

This cohort of patients took part in a mechanistic study at the National Institute for Health Research (NIHR) Imperial Clinical Research Unit Facility at Hammersmith Hospital (London, UK) from July 2016 to October 2018. See Figure 1A for patient flow through the study. Inclusion and exclusion criteria and demographic characteristics as well as data on weight, fructosamine, fasting glucose, and insulin levels at baseline and 4 weeks post each intervention have been published previously (6). Briefly, the study was a single-blind, randomized, placebo-controlled study comparing two 28-day infusion groups: tripeptide GOP or 0.9% saline vehicle (SAL) in patients with obesity and prediabetes or T2D. The weight-adjusted doses of each GOP component were 4 pmol/kg/min (GLP-1), 4 pmol/kg/min (OXM), and 0.4 pmol/kg/min (PYY), delivered for 12 h per day, starting an hour prior to breakfast and finishing after the last meal of the day. All infusion participants also received dietetic advice on healthy eating and weight loss from a qualified dietician.

Two similar nonblinded groups of patients, either undergoing RYGB surgery or following a VLCD meal replacement diet (approximately 800 kcal/day for 4 weeks), were also recruited as comparators. The participants undergoing RYGB attended the research unit for a baseline visit prior to surgery and were reviewed 2, 4, and 12 weeks postsurgery. VLCD participants attended the research unit for a baseline visit, before starting a complete meal replacement VLCD of 800 kcal/day for 4 weeks (Cambridge Weight Plan). They were reviewed by a dietician prediet and then on a weekly basis until completion.

Blood samples for metabolomic analysis were collected in the fasting state into lithium heparin tubes with no protease inhibitors, prior to and 4 weeks into each intervention. For the GOP and SAL groups, the second sample was collected on the last day of the study, at least 2 h after the initiation of the infusion. Plasma was obtained after centrifuging the blood samples at 2500 *×* g for 10 min at 4°C. Urinary samples were collected at a fasting state on the same study days as the plasma samples. All the samples were kept at −80°C before metabolomic analysis.

### Metabolomic Analysis

A total of 136 plasma and 136 urine samples from 68 subjects [see Supplementary Figure 1A (30)] were analyzed using ultraperformance liquid chromatography-mass spectrometry (UPLC-MS) and ^1^H NMR spectroscopy. Full analytical details, following previously described sample preparation, analytical, and quality control procedures (31-33), are provided in the Supplementary Material (30).

NMR and UPLC-MS assays were applied to maximize coverage of a broad range of metabolite classes including lipophilic, hydrophilic, small, and macromolecular analytes [see Supplementary Figure 1B (30)]. Specifically, UPLC-MS assays for plasma analysis were tailored for the separation of lipophilic analytes (eg, complex and neutral lipids) by reversed-phase chromatography (RPC) assay and the separation of hydrophilic analytes (eg, polar and charged metabolites) by hydrophilic interaction liquid chromatography (HILIC assay). UPLC-MS assays for urine analysis were tailored for broad small molecule coverage using RPC (SmMol RPC) and the separation of hydrophilic analytes by HILIC. When coupled to positive- and or negative-mode ionization the following data sets were produced: plasma lipid positive (lipid RPC+), lipid negative (lipid RPC−) and HILIC positive (HILIC+); urine small molecule positive (SmMol RPC+), and negative (SmMol RPC−) and HILIC positive (HILIC+). For both plasma and urine samples, a standard 1-dimensional (1D) ^1^H NMR profile experiment was acquired using the 1D-nuclear magnetic resonance nuclear Overhauser effect spectroscopy presat pulse sequence with water presaturation. For plasma, an additional spin-echo experiment using the 1D Carr-Purcell-Meiboom-Gill pre-sat pulse sequence was carried out to better visualize signals of the small metabolites.

Data from each assay was processed to include both global profiling and targeted extraction data sets. These 2 approaches are highly complementary. Global profiling provides a comprehensive analysis of all measurable metabolites in a sample, but results in data sets with multiple variables per analyte, the identities of which are typically unknown. In contrast, by targeted extraction of a predefined set of metabolites, preannotated data sets are immediately more interpretable but are limited in coverage to those metabolites in the predefined set. For UPLC-MS, targeted extraction of metabolites from each assay was performed using PeakPantheR (34) (https://github.com/phenomecentre/peakPantheR) and for NMR targeted extraction was performed using the in vitro diagnostics platform (IVDr) from Bruker Biospin (www.bruker.com) to generate plasma SmMol IVDr, plasma lipoprotein IVDr and urine SmMol IVDr data sets.

### Statistical Analysis

#### Unsupervised analysis by principal component analysis

To provide a broad overview of the data, including identification of trends, clusters, and any biological outliers, principal component analysis (PCA) was performed on the global profiling metabolomic data sets. For brevity, data sets were combined before analysis to generate a biofluid/platform-specific overview, with 4 overall groupings: plasma UPLC-MS (containing lipid RPC+, lipid RPC− and HILIC+ data sets; 3641 total variables), plasma NMR (containing the plasma standard 1D and Carr-Purcell-Meiboom-G data sets; 944 total variables), urine UPLC-MS (containing SmMol RPC+, SmMol RPC−, and HILIC+ data sets, 24 077 total variables) and urine NMR (containing the urine standard 1D data set; 585 total variables). To better visualize grouping patterns among the treatment groups and to take the time course-related changes into consideration, PCA was performed on fold-change data, where, for each variable and each individual, values were calculated as the log_2_ fold change between 4-week and baseline time points. PCA was performed on these derived data sets using the Scikit-learn Python package (35). To provide additional insight, 1-way analyses were performed between treatment groups (SAL, GOP, RYGB, and VLCD) for the scores of each PCA component using Prism 8.0 (GraphPad Software).

#### Supervised analysis by linear mixed effect modeling

To further investigate treatment group specific differences over the study time-course linear mixed effects (LME) modeling was performed on the targeted extraction data sets. Data sets were selectively combined to generate a metabolite class-specific overview, with 4 overall groupings: plasma small molecules (containing the SmMol IVDr data set and 38 of the HILIC+ metabolites; 67 total metabolites), plasma lipoproteins (containing the lipoprotein IVDr data set; 112 total metabolites), plasma lipids (containing the lipid RPC− and HILIC+ data sets and 11 of the HILIC+ metabolites; 269 total metabolites) and urine small molecules (containing SmMol IVRr, SmMol RPC+, SmMol RPC−, and HILIC+ data sets; 178 total metabolites). LME models were generated using the lme4 R software package (36) according to the formula: model <− log(variable) ~ time-point * treatment group + (1|subject). A model was generated for each variable including fixed effects for interaction between time point (baseline or 4 weeks post intervention) and treatment group (SAL, GOP, RYGB, or VLCD), allowing for subject-specific random effects. Metabolites with a false discovery rate (FDR) α < 0.05 controlled using the Benjamini-Hochberg procedure (37) were considered to be statistically significantly different between treatment groups (specifically compared to the SAL group). To investigate the influence of potential confounding/covarying factors additional models were generated according to the adapted formula: model <− log(variable) ~ time-point * treatment group + time-point: treatment group: factor + (1|subject). Factors investigated included difference (baseline to 4-week time points) in weight, fasting glucose, fasting insulin, triglycerides, and total cholesterol. As for the main model, significant metabolites were determined after adjustment of appropriate model estimates for multiple corrections by FDR control.

Subsequently, to investigate metabolic changes specifically associated with each of these clinical factors, the supervised multivariate method of partial least squares (PLS) was performed between metabolomic data sets (X) and each clinical factor (Y) in sample sets containing all groups (the full cohort) and each treatment group (SAL, GOP, VLCD, and RYGB) individually. The validity of PLS models was assessed by cross-validation and generation of the Y prediction performance statistic (Q^2^Ŷ) and permutation testing of the true model against 1000 null models with mismatched Y. Where valid models were obtained (Q^2^Ŷ > 0.15 and permutation *P*-value < 0.01), metabolites associated with Y (clinical factor) were selected empirically by comparing the true model weights against the null model weight distribution and selecting those where the true weight exceeded the null model weight distribution with *P* < 0.05.

## Results

### Tripeptide GOP Infusion Achieves Blood Glucose Control Without Significantly Altering the Circulating or Excretory Metabolome

The study design and participant disposition are summarized in Figure 1A. Baseline demographic and clinical characteristics are shown in Supplementary Table 1 (30). We previously reported that tripeptide GOP infusion led to better glucose tolerance in response to a mixed meal test but less weight loss than VLCD and RYGB (6). Individual participant changes from baseline in weight, fasting glucose, insulin, and lipid parameters from the current subanalysis, which includes 1 additional RYGB participant that had not completed follow-up in the original study, are presented in Figure 1B.

**Figure 2.**
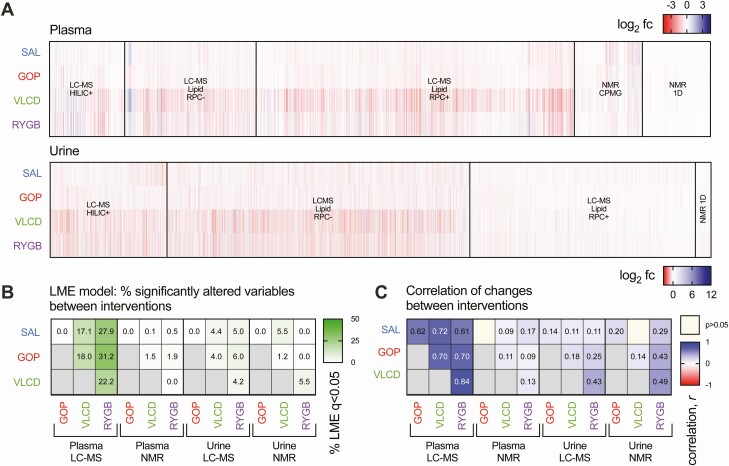
Overview of changes to untargeted plasma and urinary metabolome after each intervention. (A) Heatmap summary of profiling data from plasma and urine, represented as mean log_2_ fold change, with analytical mode indicated. NMR 1D refers to 1-dimensional nuclear magnetic resonance nuclear Overhauser effect spectroscopy 1D. (B) Summary of pairwise comparisons between interventions by linear mixed effects model, indicating the percentage of features in each data set that were statistically significantly different, with control of false discovery rate at 5% applied. (C) Correlations (Pearson’s *r*) between each intervention pair of mean log_2_ fold changes for each analytical data set.

We first performed untargeted metabolomic analysis on pre- and postintervention plasma and urine samples to identify patterns of change in an unbiased manner, without focusing on specific metabolites. It is immediately apparent from visual inspection of the metabolomic changes between pre- and postintervention timepoints (Fig. 2A) that the impact of GOP was less marked than of RYGB and VLCD and, in many cases, similar to that seen with SAL. This was further investigated using LME modeling to formally determine the proportion of changes that were significantly different between interventions (Fig. 2B). This indicated that, when compared to SAL, up to one third of the observed plasma LC-MS features were distinctly altered by RYGB or VLCD, whereas no features were significantly altered by GOP after FDR correction. However, calculating the similarity between the effects of each intervention by correlating average fold changes for each variable showed that VLCD and RYGB effects were highly congruent (Fig. 2C). In fact, even features that were statistically significantly altered only by VLCD or by RYGB still showed a high degree of correlation (ie, the directions of change, if not the magnitudes, were generally consistent) [Supplementary Figure 2A (30)]. On the other hand, urine LC-MS features identified as “uniquely” affected by VLCD or RYGB were not consistently as well correlated [Supplementary Figure 2B (30)].

As an additional analysis aiming to identify patterns in the metabolomic effects of each treatment, PCA was also performed on all recorded log_2_ fold changes. No principal components in any biofluid were significantly different between GOP and SAL, whereas VLCD and RYGB showed a number of differences when compared to SAL, GOP, and each other [Supplementary Figure 2C and 2D (30)].

This main message from this global analysis is that GOP treatment has a far more minor effect on the metabolome than VLCD or RYGB, despite being more effective for lowering blood glucose. A second message is that the metabolomic impacts of VLCD and RYGB share many common elements.

### Lipidomic Responses to VLCD and RYGB Reflect Caloric Restriction

**Figure 3.**
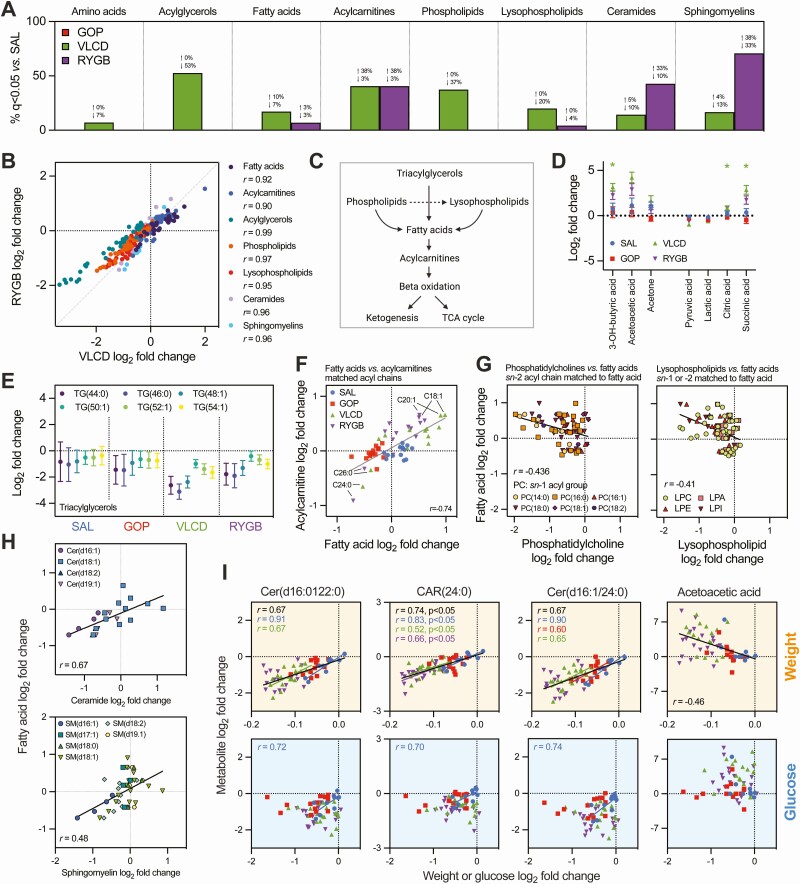
Intervention-specific changes to energetic metabolites and the plasma lipidome from preannotated data sets. (A) Percentage of species within major metabolite groups that were significantly increased or decreased by each active treatment *vs* saline (SAL). (B) Correlations (Pearson’s *r*) between mean log_2_ fold change for individual lipid species after very-low calorie diet or Roux-en-Y gastric bypass treatment; all correlations were *P* < 0.05. (C) Metabolic links between lipid subclasses and energy metabolism. (D) Intervention-specific mean log_2_ fold changes ± 95% CIs for selected energy metabolism-associated metabolites, with statistical comparisons *vs* SAL by linear mixed effects model (*q < 0.05). (E) Intervention-specific mean log_2_ fold changes ± 95% CIs for selected triacylglycerols (the minimum number of saturations available for each chain length was chosen for display). (F) Association between mean log_2_ fold changes for fatty acids and acylcarnitine species with matched acyl chains. Pearson’s *r* across the full cohort is shown. (G) As for (F) but for fatty acids *vs* phosphatidylcholine or lysophospholipid species. For phosphatidylcholines, the *sn*-2 acyl chain is matched to the fatty acid, with species with different *sn*-1 acyl chains differentiated on the graph. Each match is represented by 4 data points (1 per intervention). Pearson’s *r* for each combined data set is shown. (H) As for (G) but showing ceramides (Cer) and sphingomyelins (SM). The *sn*-2 acyl chain is matched to the fatty acid, with species with different *sn*-1 acyl chains differentiated on the graph. (I) Correlations between weight loss or change in fasting glucose and different metabolites. Pearson’s *r* is shown only where *P* < 0.05, either for the whole cohort (black) or individual groups (with corresponding color). See Supplementary Figures 3 to 6 (30) for additional correlation analysis and partial least squares modeling. Abbreviations: LPA, lysophosphatidic acid; LPC, lysophosphatidylcholine; LPE, lysophosphatidylethanolamine; LPI, lysophosphatidylinositol; PC, phosphatidylcholine.

Following this analysis of the untargeted profiling data, we next repeated the LME modeling to determine the proportion of changes that were significantly different between interventions in specific groups of metabolites from the targeted extraction data sets. Overall changes across major plasma metabolite classes relative to SAL are summarized in Figure 3A. See Supplementary Figure 1B (30) for information about the plasma and urine data sets and Supplementary Figures 3 to 7 (30) for full characterization of individual metabolites, statistical analysis of differences between groups, and associations with clinical responses. As for the untargeted data sets, no metabolites were affected by GOP compared to SAL with FDR correction (Fig. 3A). In contrast, VLCD and RYGB led to significant changes in a substantial proportion of lipid species compared to SAL (Fig. 3A). From the lipids quantified, VLCD led to a larger number of changes in acylglycerols, fatty acids, phospholipids, and lysophospholipids whereas RYGB affected ceramides and sphingomyelins to a greater extent. However, despite some variation in the statistical significance obtained for SAL-VLCD and SAL-RYGB comparisons, the lipidomic effects of these 2 interventions were very well correlated (Fig. 3B), suggesting overlap between the mechanisms driving these changes.

The pattern of lipidomic changes seen with RYGB and VLCD, characterized by decreases in acylglycerols, increases in fatty acids and acylcarnitines, but decreases in phospholipids and their lysophospholipid derivatives (Fig. 3A), is consistent with the known effects of caloric restriction (19,38). These changes reflect an energetic switch to beta oxidation, facilitated by mobilization of fatty acids from diverse sources (acylglycerols, phospholipids, and their derivatives), which are then converted to acylcarnitines for transport into mitochondria, where they undergo beta oxidation (Fig. 3C). Generation of acetyl-coenzyme A from beta oxidation provides a substrate for both ketogenesis and the tricarboxylic acid (TCA) cycles, and accordingly, elevations in ketones (eg, 3-hydroxybutyric acid and acetoacetic acid) and circulating TCA cycle intermediates (eg, citric and succinic acid) were observed, particularly after VLCD [Fig. 3D; also see Supplementary Figure 3 (30)]. Ketones in urine tended to also be increased after VLCD and RYGB [Supplementary Figure 4 (30)]. The marked reductions in triacylglycerol levels with VLCD and RYGB tended to apply particularly to shorter chain, saturated forms [Fig. 3E; also see Supplementary Figure 5 (30)], a pattern previously noted during caloric restriction (38).

Resolution of individual acyl chain carbon content in phospholipids and lysophospholipids enabled us to observe how these lipid species changed in parallel to fatty acids and acylcarnitines with the same chain length (Figs. 3F and 3G). Note that phosphatidylcholines (a major group of phospholipids) contain 2 acyl chains (*sn*-1 and *sn*-2 positions); in this case, we matched free fatty acids to the acyl chain at the *sn*-2 position, meaning there is more than 1 match for many fatty acids, with the mean change for each treatment represented by a single data point for each match (Fig. 3G, left panel). Similarly, inclusion of several different lysophospholipid subclasses (Fig. 3G, right panel) means that each fatty acid has a number of lysophospholipid matches. With these caveats in mind, it was still apparent that phospholipids and lysophospholipids tended to reduce in parallel with increases in free fatty acids of the same chain length (Fig. 3G), which is consistent with the notion that (lyso)phospholipids are a key source of fatty acids during caloric restriction (eg, sourced from cell membranes). Lysophosphatidylethanolamines and lysophosphatidylinositides showed the clearest correlations (*r* = 0.68 and 0.62, respectively) with their matched fatty acids.

While a majority of fatty acids and acylcarnitines were increased with VLCD and RYGB, we observed that the very long chain C24:0 (lignoceric acid) or C26:0 (cerotic acid) species tended to be reduced [Fig. 3F; also see Supplementary Figures 5 and 6 (30)]. The divergent effects on these very long chain fatty acids *vs* shorter chain counterparts may be a feature of caloric restriction (19,38) and are compatible with the observation that reductions in triglycerides seen with VLCD and RYGB applied preferentially to species with a lesser carbon content (Fig. 3E), as these are presumably unlikely to contain very long acyl chains.

Showing the opposite pattern to that observed with phospholipids, ceramide, and sphingomyelin species across the entire cohort tended to increase or decrease in tandem with fatty acids or acylcarnitines sharing the same acyl chain length (Fig. 3H). C24:0- and C26:0-containing sphingomyelins and ceramides were well represented in our preannotated lipidomic panel but not in the phospholipid or lysophospholipid panels; due to the contrasting direction of change seen with these very long chain species compared to their shorter counterparts, this could explain why the effects of RYGB and VLCD on sphingomyelin/ceramide lipids appeared (somewhat artefactually) more heterogenous than for phospholipids/lysophospholipids (Fig. 3A).

We also investigated the relationship between changes in the previously mentioned lipids or energetic metabolites and changes in clinical parameters including weight, fasting glucose, insulin, triglycerides, and cholesterol across the whole cohort and within individual interventions. Approximately 60% of quantified lipids showed a significant correlation with weight loss across the entire cohort, with a smaller number (~23%; mainly fatty acids) significantly correlated with glucose, and 56%, 44%, and 40%, respectively, correlated with changes in insulin, cholesterol, and triglycerides [Supplementary Figures 5 and 6 (30)]. However, using LME modeling, owing to the observed group-specific differences in these clinical parameters (Fig. 1B), it is hard to mathematically separate the impact of treatment from the impact of differences in clinical values on the metabolome. This was therefore further investigated using PLS analysis by building models between the metabolomic data and each clinical parameter across all samples (ie, all treatment groups combined) and separately for individual interventions. Interestingly, even where valid models were obtained, for the majority of lipids the observed associations did not meet an FDR-corrected *P*-value threshold of 0.05 [Supplementary Figures 5 and 6 (30)]. Those lipid species that did show a statistically significant association with weight loss (but not glucose) across the whole cohort included CAR(24:0) and selected ceramide, sphingomyelin, and phosphatidylcholine species. Of these however, only the 2 ceramide species (d16:1/22:0 andd16:1/24:0) showed any associations with weight loss when individual groups were modeled separately (Fig. 3I); significant Pearson’s correlations with blood glucose were seen for these lipids in the SAL group, but these were not significant by PLS modeling [Supplementary Figures 5 and 6 (30)]. Acetoacetic acid remained predictive of weight changes across the entire group (Fig. 3I).

In summary, we observed that VLCD and RYGB led to marked and well-correlated changes in several lipid parameters that can be explained by caloric restriction. A relationship between some of these changes and weight loss was observed. The effects of GOP on the lipidome were minor.

### Effects on Amino Acid Metabolism

**Figure 4.**
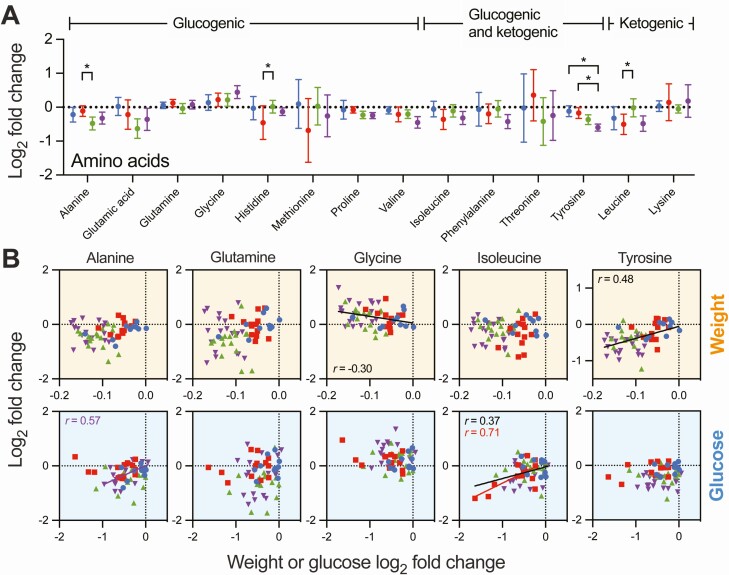
Effects on amino acid metabolism. (A) Intervention-specific mean log_2_ fold changes ± 95% CIs for plasma amino acids, with statistical comparisons between groups by linear mixed effects model (*q < 0.05). (B) Correlations between weight loss or change in fasting glucose and selected amino acids. Pearson’s *r* is shown only where *P* < 0.05, either for the whole cohort (black) or individual groups (with corresponding color). See Supplementary Figures 3 and 4 (30) for additional correlation analysis and partial least squares modeling.

Caloric restriction can affect amino acid metabolism, in part as several amino acids serve as substrates for gluconeogenesis, ketogenesis, or both (19,38,39). In our study, only 1 amino acid (tyrosine) was significantly altered by an active treatment (RYGB) compared to SAL treatment [Fig. 3A; also see Supplementary Figure 3 (30)]. However, there were a number of other statistically significant intergroup comparisons and nonsignificant but informative trends [Fig. 4A; also see Supplementary Figure 3 (30)]. For example, with VLCD and RYGB, trends toward reductions in alanine and glutamic acid, and increases in glycine were observed. This pattern is reported in the context of starvation (38,39), with the drop in alanine levels thought to represent increased alanine utilization as a gluconeogenic substrate, thereby fitting with reduced levels of pyruvic acid (another gluconeogenic substrate) after VLCD (Fig. 3D). Reductions in blood glucose for the RYGB group, but not weight loss, were associated with greater reductions in alanine in the PLS [Fig. 4B; also see Supplementary Figure 3 (30)]; this did not apply to other interventions or across the cohort as a whole. Changes in glycine were inversely associated with weight loss across the whole cohort by Pearson’s correlation (Fig. 4B), but this was not significant by PLS modeling for the entire group or for individual interventions [Supplementary Figure 3 (30)]. The fact that alanine and glycine, both considered gluconeogenic amino acids, showed divergent changes with VLCD indicates that the effects of caloric restriction on amino acid metabolism cannot be considered only within the framework of fuel utilization.

RYGB has previously been associated with a specific lowering effect on BCAAs, with this effect predictive of improvements in glucose homeostasis (23). Notably, in our study, all 3 BCAAs (valine, leucine, isoleucine) were numerically decreased, albeit nonsignificantly, after RYGB compared to SAL (Fig. 4A), whereas for VLCD there was no change. Interestingly, isoleucine was also reduced after GOP treatment compared to baseline, although this was driven primarily by 3 participants who showed quite marked reductions in this BCAA (Fig. 4B). The same 3 GOP participants also showed the greatest reductions in blood glucose, and accordingly, changes in isoleucine were correlated with changes in blood glucose across the whole cohort (Fig. 4B). This was the case for leucine [Supplementary Figure 3 (30)], and the glucose-isoleucine association within the GOP treatment group remained significant in the PLS model [Supplementary Figure 3 (30)]. The effects of RYGB and GOP on BCAAs are unlikely to be explained by caloric restriction, which is known to cause an initial increase in circulating BCAAs and a subsequent decline if starvation is prolonged over many days (38,39). Compared to VLCD, for which the metabolomic effects of caloric restriction are most pronounced (Fig. 3), consistently lower BCAAs in the RYGB group are suggestive of a distinct mechanism unrelated to caloric restriction.

The RYGB-mediated reduction in plasma tyrosine (Fig. 4A) was associated with weight loss across the entire cohort, both by Pearson’s correlation (Fig. 4B) and by PLS analysis [Supplementary Figure 3 (30)]. Urinary tyrosine was also significantly reduced after RYGB *vs* SAL [Supplementary Figure 4 (30)], similar to a previous study of sleeve gastrectomy (40).

These data therefore reflect the effects of caloric restriction on amino acid metabolism, along with superimposed intervention-specific effects, mainly of RYGB, apparently achieved via a different mechanism.

### Identification of Additional VLCD- and RYGB-Discordant Metabolite Changes

Analysis of both global profiling and preannotated data sets thus far highlight many similarities between the effects of VLCD and RYGB, in many cases (especially for lipid species) explicable by caloric restriction. On the other hand, differences in plasma and urine amino acid responses indicate these interventions are not entirely equivalent at the metabolomic level (Fig. 4). We therefore sought to identify additional metabolites showing differential responses between RYGB and VLCD, focussing on the nonlipid small molecule preannotated data sets in plasma and urine (the corresponding analysis for plasma lipids is already shown in Figure 3B and shows a high degree of correlation).

**Figure 5.**
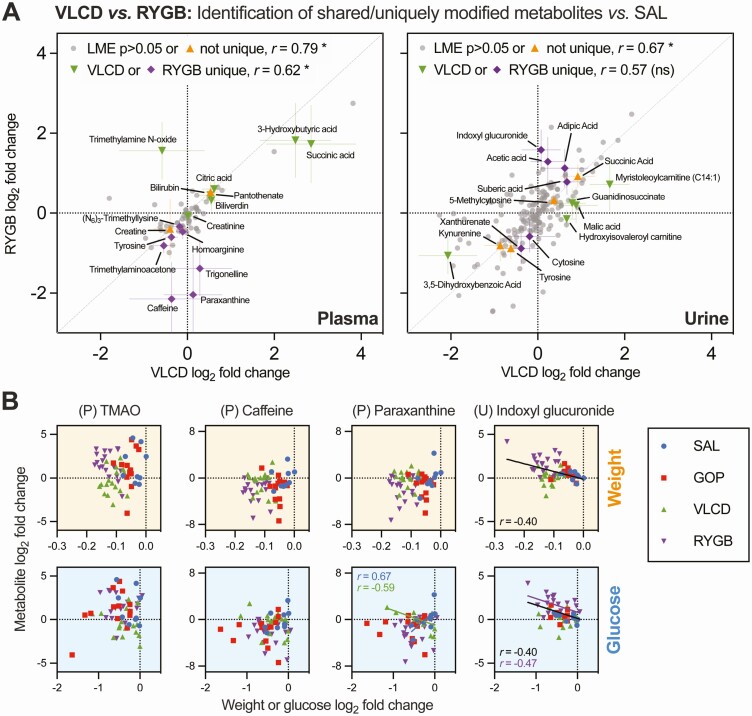
Intervention-specific changes to non-lipid small molecule plasma metabolites from pre-annotated data sets. (A) Scatter plots indicating the relationship between very-low calorie diet (VLCD) and Roux-en-Y gastric bypass (RYGB) effects (mean log_2_ fold change *vs* baseline) on plasma and urine metabolites; Pearson’s *r* between VLCD and RYGB effects are reported for nonsignificant plus jointly affected metabolites (“not unique”) *vs* for metabolites “uniquely” affected by 1 intervention only, as identified by the linear mixed effects model. 95% CIs are shown for significantly affected analytes. (**B**) Associations between weight loss or change in fasting glucose and selected plasma (P) or urine (U) metabolites identified as VLCD/RYGB discordant in (A). Pearson’s *r* is shown only where *P* < 0.05, either for the whole cohort (black) or individual groups (with corresponding color).

We identified metabolites that were significantly affected by either VLCD or RYGB, compared to SAL, and categorized these changes as “uniquely significant” to 1 intervention or “shared” (Fig. 5A). A number of metabolites, such as plasma pantothenate, plasma creatine, urine succinic acid, and urine kynurenine, showed concordant responses (ie, both significantly altered compared to SAL and both in the same direction). Other metabolites clearly showed similar direction of change but were only significant for 1 intervention compared to SAL (eg, plasma 3-hydroxybutyric acid, plasma citric acid, and urine suberic acid). We interpret these as reflecting a lack of statistical power rather than true intervention-specific changes. However, a smaller number of metabolites were identified that did not show a consistent pattern between interventions. For example, plasma caffeine and its metabolites trigonelline and paraxanthine were all decreased with RYGB but unchanged with VLCD (Fig. 5A), with trends in the same direction for the same metabolites (and also theophylline) in urine [Supplementary Figure 4 (30)]. Plasma trimethylamine-*N*-oxide (TMAO) was increased with RYGB and slightly decreased with VLCD (Fig. 5A); this effect of RYGB has been described on other occasions (41,42). Urine indoxyl glucuronide, a metabolite of tryptophan, was increased with RYGB but unchanged with VLCD (Fig. 5A). This may reflect accelerated tryptophan metabolism as urinary tryptophan was particularly reduced with RYGB [Supplementary Figure 4 (30)]. Of these highlighted metabolite changes, only urinary indoxyl glucuronide showed an association with changes in clinical parameters (weight, fasting glucose) across the entire cohort (Fig. 5B), but this was not significant by PLS modeling [Supplementary Figure 4 (30)].

### Intervention-specific Effects on Circulating Lipoproteins and Their Lipid Cargo

**Figure 6.**
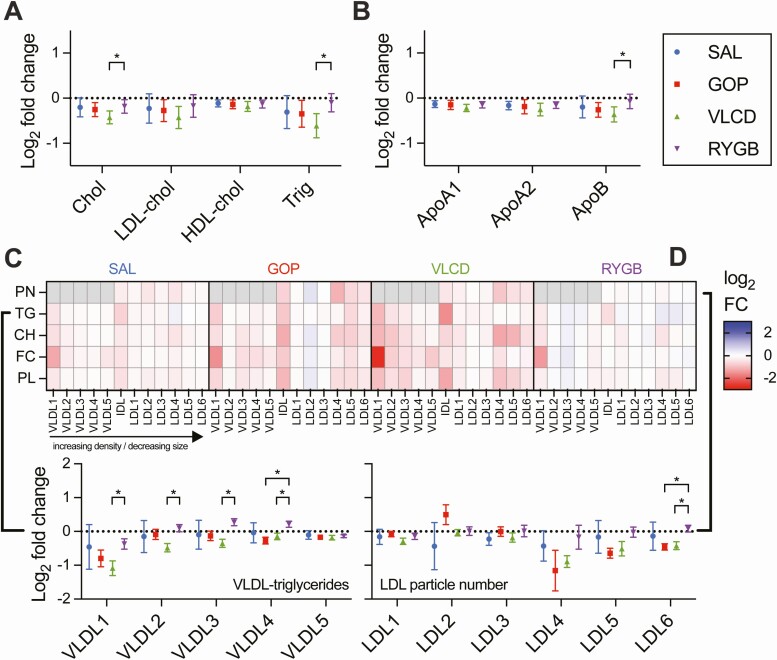
Intervention-specific changes to plasma lipoproteins. (A) Intervention-specific mean log_2_ fold changes ± 95% CIs for total cholesterol (Chol), low-density lipoprotein-associated cholesterol (LDL-chol), high-density lipoprotein-associated cholesterol (HDL-chol), and triglycerides, as determined by nuclear magnetic resonance. Statistical significance determined by linear mixed effects (LME) model, with control of false discovery rate (FDR) at 5% applied. (B) As for (A) but showing apolipoprotein A1 (ApoA1), apolipoprotein A2 (ApoA2), and apolipoprotein B100 (ApoB100). (C) Heatmap representation of mean log_2_ fold changes for apoB-containing subfraction parameters, arranged in order of decreasing size/increasing density, including particle number (PN) and content; the latter includes triglycerides (TG), cholesterol (CH), free cholesterol (FC) and phospholipids (PL). Very low-density lipoprotein (VLDL) and intermediate-density lipoprotein (IDL) fractions, as well as LDL, are shown. Also shown are same data for LDL particle number and VLDL triglycerides, but including 95% CIs and statistical significance as determined by LME model, with control of FDR at 5% applied. *P < 0.05.

In the original description of this trial, minor changes only were observed in the standard clinical lipid profile after 4 weeks of each intervention, with the most notable effects being a reduction in triglycerides with VLCD and a paradoxical increase with RYGB, compared to SAL (6). This pattern was confirmed using NMR lipoprotein analysis, which is able to subclassify a large number of lipoprotein subfractions and their lipid content [Fig. 6A; also see Supplementary Figure 7 (30)]. VLCD also reduced total apolipoprotein B100 (apoB), the protein compound of very low-density lipoprotein (VLDL) and low-density lipoprotein (LDL), whereas RYGB did not (Fig. 6B). Examining the abundance and content of differently sized apoB-containing lipoprotein subfractions revealed a similar pattern for both GOP and VLCD, in which particle number and carriage of triglycerides, cholesterol, free cholesterol, and phospholipids on the smaller, denser LDL subfractions (LDL4-6) tended to be reduced (albeit not significantly compared to SAL), while the larger, lighter subfractions (LDL1-3) were less affected (Fig. 6C). Moreover, the effect of VLCD on VLDL-associated lipids particularly affected the very lowest density subfraction (VLDL1) (Fig. 6C), for which insulin is a key regulator of production (43), and thus improvements in insulin sensitivity may be expected to exert a prominent effect. Although no lipoprotein-related differences passed adjustment for weight loss [Supplementary Figure 7 (30)], no valid models were obtained for PLS between the lipoprotein data and weight (either across all groups or for individual groups), perhaps indicating that these changes are indeed treatment-associated rather than weight loss–associated.

## Discussion

This study provides a comprehensive profiling and comparison of the plasma and urine metabolomic changes across 3 different active interventions (ie, GOP, VLCD, RYGB) for weight loss and diabetes improvement, along with a placebo control group (SAL). The effects of the tripeptide gut hormone infusion on the metabolome have not previously been investigated but are of significant clinical interest given the current drive to develop pharmacological agonists that target multiple gut hormone receptors (44). VLCD and RYGB have been subjected to several metabolomic investigations in the past, although in many cases without a suitable control group. The potential role of gut hormones in mediating metabolic effects of RYGB has been often discussed, so our direct comparison of these two interventions within the same study provides an opportunity to evaluate this hypothesis. The 2 main messages from our study are that (1) GOP treatment results in very few changes to the global metabolome yet exerts a powerful glucose-lowering effect, and (2) RYGB and VLCD exert profound metabolomic changes, which are in many cases well correlated, although with a number of exceptions as outlined in more detail in the following discussion.

Our original study showed that the glucoregulatory effects of the tripeptide GOP combination exceed those of VLCD and RYGB despite lesser weight loss (6). The individual components of the tripeptide treatment [ie, GLP-1 (7-36) amide, OXM, and PYY(3-36)] are known to exert distinct effects on glucose and energy homeostasis. GLP-1 stimulates insulin secretion, slows gastric emptying, and suppresses appetite (45). OXM, through its joint targeting of GLP-1 and glucagon receptors, not only reinforces the effects of GLP-1 itself but also increases energy expenditure, enhancing achievable weight loss (46). PYY(3-36) is primarily known for its anorectic action and has no significant acute effects on insulin secretion and sensitivity (47,48). To our knowledge, neither OXM nor PYY have been evaluated for their effects on the metabolome in the clinical or preclinical setting, although some clues into the anticipated effects of OXM are provided by an earlier study demonstrating glucagon-induced suppression of plasma amino acid levels to provide fuel for gluconeogenesis (49) and by a more recent study in mice showing that the dual glucagon/GLP-1 receptor agonist cotadutide modifies the amino acid and lipid hepatic metabolome (50).

Some studies of the effects of pharmacological GLP-1R agonists on metabolomic parameters have been reported and provide context to our observations with GOP tripeptide. Specifically, treatment with the GLP-1R agonist liraglutide over a similar time period to our study led to a reduction in small dense LDL particles (29), a trend also seen in our GOP cohort. This profile is typically associated with reduced atherogenic potential and is in keeping with the known cardiovascular benefits of GLP-1R agonist treatment (51). One year of liraglutide treatment resulted in reductions in total apoB (28), which was not the case with GOP in our study. Women with polycystic ovarian syndrome treated with exenatide for 3 months showed a reduction in BCAAs compared to baseline (52); we also observed a trend for a reduction in BCAAs with GOP, although this was not statistically significant compared to SAL.

However, while some metabolically plausible trends were seen with the tripeptide combination, it is important to highlight that that not a single plasma or urinary metabolite in either the profiling or preannotated data sets reached statistical significance with GOP compared to SAL after correction for false discovery. This may be partly due to the relatively small sample sizes in our study and the observed subtle degree of weight loss in the SAL group “diluting” the potential for observing differences with the GOP group. We performed a power analysis using the SAL group as a control by simulating the effect of increasing the size of the GOP group to that of the RYGB group on power to detect metabolomic changes [Supplementary Methods and Supplementary Figure 8 (30)]; this indicated that the slight differences in sample size were unlikely to explain the large changes in number of metabolomic changes observed; that is, the metabolomic effects of GOP are almost certainly more minor than for VLCD and RYGB, despite outperforming these 2 interventions for anti-hyperglycemic effect. Whether this glucocentric action of tripeptide treatment represents a therapeutic advantage or disadvantage is not clear, but it may hold some relevance to pharmacological approaches targeting the same receptors. Notably, the on-infusion steady state plasma concentration of GLP-1 achieved in the GOP cohort were comparable to the calculated free concentration of semaglutide in humans (53), although there are no equivalent comparisons available for OXM or PYY. On the other hand, GOP was administered as a 12-h infusion with an overnight break, contrasting with the extended pharmacokinetic profiles of long-acting GLP-1R agonists. The potential for GLP-1R tachyphylaxis to influence metabolic responses (54,55) should therefore be borne in mind when extrapolating our findings to the pharmacological setting.

The effects of bariatric surgery and dietary manipulation have been subject to intense investigation using metabolomic approaches (22). A key area of controversy is how much of the weight loss and metabolomic perturbation seen with bariatric surgery is due to caloric restriction *vs* specific physiological changes (eg, the gut microbiota) induced by the procedure. The effects of acute (48-h) caloric restriction have been comprehensively documented (19) and include increased generation of ketones and ketone derivatives, increased flux through the TCA cycle leading to increases in circulating TCA cycle intermediates, and increased lipolysis resulting in reduced acylglycerols but increases in fatty acids and acylcarnitines, as well as reductions in most phospholipids and lysophospholipids, also sources of free fatty acids destined for beta oxidation. All of these patterns have been described for RYGB and diet-induced weight loss (12,23) and were observed in our RYGB and VLCD cohorts.

Notably, some phospholipid species that were not decreased by acute caloric restriction in the study by Collet et al (19) were also conspicuously unaffected by RYGB and VLCD in our study, including PC(16:0/16:0), PC(16:0/22:6), and PE(16:0/20:4). This pattern was not universally the case though; for example, PC(16:0/20:4) was decreased by RYGB and VLCD in our study but increased with acute caloric restriction (19). In contrast, while Collet et al found increases in several sphingomyelins (19), we observed a diversity of impacts on both sphingomyelins and ceramides, with some species increased [e.g., SM(d18:1/18:0)] and some decreased [e.g., SM(d16:0/22:0)]. This may be due to having measured different sphingomyelin species in the different studies, and indeed, our preannotation pipeline identifies several examples of ceramides or sphingomyelins containing very long chain (C22:0+) acyl groups, which, as previously highlighted, behaved somewhat differently to shorter chain fatty acids. Alternatively, this discrepancy could indicate adaptation to the different intervention durations.

Nevertheless, an important message from both the global profiling data and our preannotated data sets was that both VLCD and RYGB effects were often very well correlated, even if the effect magnitudes differed in some cases, implying at least some common underlying mechanisms driving these changes. This interpretation is consistent with the conclusions of Herzog et al, who demonstrated that several metabolomic changes commonly attributed to bariatric surgery are also seen with presurgical dietary restriction in a longitudinal study which examined the metabolomic changes in patients after undergoing a 4-week preoperative VLCD prior to RYGB (27). We point out here again that our RYGB group did not undergo a preoperative VLCD; that is, the metabolomic similarities between RYGB and VLCD are not confounded by the imposition of a VLCD during preoperative preparation.

On the other hand, in the context of somewhat congruent metabolomic impacts of RYGB and VLCD, the smaller number of discrepant analytes are of particular interest as they provide more insights into physiological differences of each intervention. For example, caffeine and its metabolites trigonelline and paraxanthine were markedly decreased in our RYGB cohort, which was not the case after VLCD. In our study, all participants were requested to refrain from caffeine consumption for 24 hours before sample collection. A reduction in paraxanthine after caffeine loading in post-RYGB patients was previously reported (56), which was attributed to the effects of the surgical procedure on hepatic cytochrome P450 enzyme induction (57). Of broader relevance, RYGB surgery profoundly alters the gut bacterial composition (eg, increased *Gammaproteobacteria*) and metabolic functions (eg, increased protein putrefaction) (58,59), with the latter reflected by increased urinary host-bacterial metabolites following RYGB, such as 4-cresyl sulfate, 4-hydroxyphenylacetate phenylacetylglutamine, and indoxyl sulfate, the precursors of which are tyrosine, phenylalanine, and tryptophan (60). Thus, the significantly lower levels of plasma tyrosine, together with a trend toward lower phenylalanine, in the RYGB group compared to SAL or GOP could result from the incomplete digestion of protein and absorption of these amino acids in the foregut postsurgery. As a result, partly digested peptides could be more abundant in the colon as substrates for bacterial putrefaction. Consistent with these observations, we found an increased urinary concentration of indoxyl glucuronide after RYGB, suggesting a higher degree of bacterial conversion of tryptophan to indole and subsequent conjugation with glucuronide in the liver. Moreover, we observed relative elevations in TMAO after RYGB compared to VLCD, in line with other studies and thought to depend on the effect of the procedure on the gut microbiota (61).

There are several limitations of our study. Firstly, our postintervention sample was collected at 4 weeks, meaning we were unable to examine the sustainability of changes and how these could potentially impact on the longer-term effects of each intervention. Studies with serial sampling have demonstrated that some of the changes seen after bariatric surgery are transient (62). While in keeping with the sample sizes of analogous interventional metabolic studies, the relatively small number of participants in the SAL and GOP groups may have reduced our ability to robustly identify small but important metabolomic changes, especially when investigating the potential influence of confounding/covarying clinical factors such as weight loss. Our use of preannotation and semiautomated feature identification markedly streamlines the analytical pipeline compared to traditional approaches, facilitating biological insights by documenting changes in specific analyte groups. However, this strategy is dependent on the analytes included in the preannotated panel, which risks interpretative bias. Therefore, the global profiles provided in the current study are complementary to the preannotated data sets. An important point when interpreting metabolomic studies is that a large number of metabolites reflect recent dietary intake (63), meaning that care must be taken not to overinterpret changes within individuals. While this frequently can be a random nuisance factor, in our study changes to dietary habits are, to varying extents, inherent to the intervention. Patients consumed set calorie-restricted meals as part of the VLCD, and strict postprocedure dietary changes in the RYGB group are required as part of postoperative recovery. Changes to food preference with bariatric surgery, and indeed with GLP-1 treatment, are well recognized (64). As a further limitation, while our study benefits from analysis of both circulating (plasma) and excretory (urine) metabolome, a more comprehensive description of the intervention-specific changes could be provided by including additional analyses such as of the faecal metabolome and microbiome. Indeed, the use of GLP-1R agonists has been associated with significant changes to the intestinal microbiome (65), and, in reverse, the microbiome may modulate responses to GLP-1R agonist treatment (66). Finally, the descriptive nature of this study is inherently hypothesis-generating rather than mechanistic; further experimental work will be required to unveil the role of metabolomic changes described herein.

In conclusion, our study provides a comprehensive profiling of the metabolomic phenotypes of 3 different interventions for weight loss and T2D. We have shown that the effects of GOP on the metabolome are far more minor than for VLCD and RYGB. Overall, tripeptide GOP treatment has more powerful effects on glucose than VLCD and RYGB but does not replicate the more profound effects of VLCD and RYGB on the metabolome, the duration, and mechanistic importance of which are still to be fully elucidated.

## Data Availability

The data generated and analyzed during this study is available upon reasonable request from the authors.

## Additional Information

***Disclosure Summary:*** The authors affirm there are no competing interests relevant to this project.
